# Metabolic Reprogramming in Breast Cancer and Its Therapeutic Implications

**DOI:** 10.3390/cells8020089

**Published:** 2019-01-26

**Authors:** Nishant Gandhi, Gokul M Das

**Affiliations:** Department of Pharmacology and Therapeutics, Center for Genetics & Pharmacology, Roswell Park Comprehensive Cancer Center, Buffalo, NY 14263, USA; nishant.gandhi@roswellpark.org

**Keywords:** breast cancer, metabolism, estrogen receptors, p53, standard-of-care, resistance mechanisms, molecular subtypes, tumor microenvironment, mitochondria, mito-nuclear crosstalk, metabolism in metastatic cascade, metabolic reprogramming, precision medicine

## Abstract

Current standard-of-care (SOC) therapy for breast cancer includes targeted therapies such as endocrine therapy for estrogen receptor-alpha (ERα) positive; anti-HER2 monoclonal antibodies for human epidermal growth factor receptor-2 (HER2)-enriched; and general chemotherapy for triple negative breast cancer (TNBC) subtypes. These therapies frequently fail due to acquired or inherent resistance. Altered metabolism has been recognized as one of the major mechanisms underlying therapeutic resistance. There are several cues that dictate metabolic reprogramming that also account for the tumors’ metabolic plasticity. For metabolic therapy to be efficacious there is a need to understand the metabolic underpinnings of the different subtypes of breast cancer as well as the role the SOC treatments play in targeting the metabolic phenotype. Understanding the mechanism will allow us to identify potential therapeutic vulnerabilities. There are some very interesting questions being tackled by researchers today as they pertain to altered metabolism in breast cancer. What are the metabolic differences between the different subtypes of breast cancer? Do cancer cells have a metabolic pathway preference based on the site and stage of metastasis? How do the cell-intrinsic and -extrinsic cues dictate the metabolic phenotype? How do the nucleus and mitochondria coordinately regulate metabolism? How does sensitivity or resistance to SOC affect metabolic reprogramming and vice-versa? This review addresses these issues along with the latest updates in the field of breast cancer metabolism.

## 1. Introduction

As an emerging hallmark of cancer, altered metabolism has gained significant traction for therapeutic intervention over the last decade. The emergence of this field stemmed from a seminal observation made by Dr. Otto Warburg that cancer cells, unlike normal cells, preferentially undergo glycolysis in the presence of an oxygen rich environment [1]. This phenomenon, termed the Warburg effect, was one of the first indicators of a metabolic vulnerability observed in cancer cells. With the advent of new technologies, a number of recent studies have reiterated the importance of metabolic reprogramming in various cancers. While the importance of glycolysis in the survival and progression of certain cancers is undeniable, it has increasingly become evident that cancer cells may preferentially utilize a number of alternative metabolic pathways to drive their phenotype [2,3]. Interestingly, recent reports suggest that cancer cells may use one or multiple metabolic pathways depending on their stage in the metastatic cascade [4]. Furthermore, cancer cells may adopt a specific metabolic program based on the site to which they metastasize [5,6,7,8]. Both cell extrinsic and intrinsic cues are believed to govern the type of metabolic program adopted by a cancer cell [9]. 

Altered metabolism is a characteristic feature of both treatment-naive and treatment-resistant breast cancer. The current treatment options for breast cancer depend on the broad classification of breast cancer into (i) estrogen receptor α (ERα)-positive, progesterone receptor (PR)-positive; (ii) human epidermal growth factor receptor 2 (HER2) enriched, and (iii) triple negative breast cancer (TNBC) that do not express any of these three receptors [10,11,12,13]. About 70% of the breast cancer cases present as ERα+ and are treated with hormone therapy [12]. HER2 overexpressing cancers occur in 25–30% of the cases and are frequently treated with Trastuzumab [14,15]. There is no targeted therapy for TNBC that constitutes 15–20% of breast cancer cases and therefore, conventional cytostatic chemotherapy remains the only therapeutic option. Treatment failure in all these breast cancer types occurs as a consequence of inherent or acquired resistance [16,17]. One of the ways cells become resistant to therapy is by rewiring their metabolism; therefore, these metabolic alterations can potentially be exploited for therapeutic intervention. The combination of metabolic intervention along with the above-mentioned standard-of-care (SOC) treatment has been investigated but the results have been mixed. While significant strides have been made in this area of research, several questions still warrant deeper investigation. What are the metabolic differences between the different subtypes of breast cancer? Do cancer cells have a metabolic pathway preference based on the site and stage of metastasis? How do the cell intrinsic and extrinsic cues dictate the metabolic phenotype? How does crosstalk between the nucleus and cytoplasm/mitochondria regulate metabolism? How does sensitivity or resistance to SOC affect metabolic reprogramming and vice-versa? This review attempts to address these important issues while summarizing the developments made in the field of altered metabolism in breast cancer.

## 2. Determinants of Metabolic Reprogramming

Several factors contribute towards driving metabolic reprogramming in cancer cells. These can be broadly classified into cell-intrinsic and -extrinsic cues. Intrinsic cues include oncogenes and tumor suppressor genes that regulate metabolic pathways at multiple levels in different cellular compartments. There are several regulators of breast cancer metabolism such as phosphatidylinositol-4,5-bisphosphate 3-kinase (PI3K), MYC, ER, breast cancer susceptibility gene 1 (BRCA1), and p53 [18,19,20,21,22,23,24,25,26,27,28,29]. Furthermore, there is functional interplay between MYC, ERα, BRCA, and p53 [30,31,32,33]. This review focuses on how ER and p53 intricately regulate breast cancer metabolism on their own or via inter-dependent mechanisms including protein–protein interactions. 

In addition to the cell-intrinsic cues, cell-extrinsic cues comprise of nutrient availability, hypoxia, acidosis and the interaction of the tumor with components of its microenvironment (TME), including surrounding blood vessels, immune cells, fibroblasts, bone marrow-derived inflammatory cells, lymphocytes, and the extracellular matrix. These factors create a selection pressure on cancer cells, where only those cells that undergo a favorable metabolic transformation survive. The changes in metabolic pathways/fluxes induced by either extrinsic or intrinsic factors often impinge on each other providing avenues for several feedforward and feedback loops. Both of these factors are discussed in greater detail in the following sections.

### 2.1. Cell Intrinsic Cues: Role of Estrogen Receptors and p53 in Breast Cancer

Estrogen receptors alpha and beta (ERα and ERβ) play a critical role in mediating the development and maturation of the normal mammary gland. The two estrogen receptors vary significantly in their tissue distribution. While ERα is predominantly expressed in the nuclei of epithelial cells, ERβ on the other hand has been reported to be expressed in both cytoplasmic and nuclear compartments of a variety of cells [34]. ERβ’s expression has been reported in luminal epithelial cells, myoepithelial cells, intralobular stromal cells, endothelial cells lining blood vessels as well as in lymphocytes. In the normal mammary gland, ERβ’s expression is far more widespread than ERα [35]. Studies involving the genetic deletion of these receptors in mice have reported that ERβ^−/−^ but not ERα^−/−^ undergo normal mammary gland development, highlighting the importance of ERα in this process [36]. Although early studies showed that mice that were homozygous null for p53 had overall normal development [37,38] and were normal in mammary gland involution and remodeling [39], p53 has been shown to play a critical role in regulating mammary stem cell properties such as self-renewal and differentiation [40,41]. Notably, a delay in apoptosis and involution was observed in p53^−/−^ mice compared to mice bearing wild-type p53 [42]. When the effect of p53 dosage on mammary gland development was analyzed utilizing the p53^+/m^ virgin mouse, defect in mammary gland ductal morphogenesis was observed. This phenotype was rescued by the induction of pregnancy or treatment with estrogen, progesterone, combination of both, or IGF1 [43].

A number of studies have reported p53 playing a tumor suppressive role in mammary tumorigenesis [44,45,46,47]. Furthermore, several epidemiological studies have shown a reduced risk of breast cancer in women who underwent full-pregnancy early in their lives [48,49,50,51]. These observations were further validated in several in vivo models [52,53,54,55,56]. Subsequently, it was shown that treatment with the combination of estrogen and progesterone or human chorionic gonadotrophin mimicked the protection to a carcinogen challenge similar to early stage, full-term pregnancy as mentioned above [55,57,58]. Elegant studies have shown p53 to play a crucial role in mediating hormone-induced refractoriness to carcinogen-induced breast cancer [45,56]. Together, these studies showed that hormone treatment induced a sustained induction of nuclear localization of functional p53 and that loss of p53 abrogated the hormone-induced refractoriness to breast cancer.

#### 2.1.1. Role of ERα in Regulating Breast Cancer Metabolism

Adipose tissue is abundant in the breast tumor microenvironment. Hormones, growth factors and adipokines (a heterogeneous group of signaling molecules) influence the growth and progression of breast tumors [59,60]. It is important to understand the role of ERα in regulating the metabolism of the adipose tissue when studying breast cancer as the interactions between the adipocytes and the surrounding mammary epithelial cells can have significant metabolic consequences. 17β-estradiol (E2) increases the expression of insulin receptors and decreases the lipogenic activity of lipoprotein lipase in the adipose tissue [61]. Ovariectomized rats display high levels of fasting glucose and insulin, decreased phosphorylation of adenosine monophosphate kinase (AMPK) and acetyl-CoA carboxylase (ACC) in the adipose tissue. Genes involved in glucose homeostasis such as peroxisome proliferator-activated receptor coactivator-1 α (PGC-1 α), adiponectin and uncoupling protein 2(UCP2) were decreased, while levels of resistin were increased. Hypoglycemia was reverted to normoglycemia on treatment with E2 with concomitant inversion of expression levels of the genes mentioned above [62]. In mice, adipose triglyceride lipase signaling was increased on ovariectomy that was subsequently attenuated on treatment with E2 [63]. The administration of tamoxifen to ERβ^−/−^ mice significantly decreased glucose transporter 4 (GLUT4) expression indicating that ERα regulates GLUT4 in the adipose tissue [64]. 

Adiponectin, one of the adipocytokines secreted by the adipose tissue, has an important role in obesity-associated breast cancer [65]. AMPK, a key protein in adiponectin signaling is activated by LKB1. Recently, it has been reported that ERα-LKB1 interaction negatively interferes with AMPK phosphorylation by LKB1 leading to inhibition of TSC2/mTOR/p70S6k signaling [66]. Importantly, while adiponectin inhibits AMPK activity in ERα-positive MCF-7 cells, it activates AMPK in the MDA-MB-231 TNBC cells. Therefore, the anti-tumorigenic/anti-metastatic effect of adiponectin appears to be dependent on the ERα status of breast cancer. 

E2 and ERα have also been shown to reprogram metabolism based on glucose availability. In the presence of high levels of glucose, it has been reported that E2 enhances glycolysis while repressing the tricarboxylic acid (TCA) cycle activity. When glucose levels are low, E2 stimulates the increased utilization of the TCA cycle to meet the energy needs of the cancer cell [67]. ERα has also been shown previously to mediate the transcription of hypoxia inducible factor-1α (HIF-1α) which is upregulated at times of hypoxic stress. HIF-1α has also been previously reported to induce a glycolytic signature of metabolic genes and hence, ERα can indirectly activate glycolysis via the activation of HIF-1α [68].

In addition to their nuclear functions, ERs and E2 also play an important role in the mitochondria. The mitochondrial electron transport chain comprises of several complexes formed by proteins that are encoded by the nuclear or mitochondrial genome. ERα and E2 have been reported to be involved in regulating transcription of both nuclear and mitochondrial genes, encoding proteins that are important for mitochondrial functioning. This will be discussed in further detail later in the review. 

#### 2.1.2. Role of ERβ in Regulating Breast Cancer Metabolism

The pioneering discovery of ERβ almost 20 years ago [69,70,71,72,73] led to a reevaluation of the actions mediated by E2. ERβ and ERα are encoded by separate genes; however, they bear a significant similarity in their structure and function. ERβ’s maximal sequence homology to ERα lies in its DNA binding domain (DBD: 96%) and the least similarity in its transactivation domain (TA: 30%). The two receptors also share a 53% sequence homology in their ligand-binding domain (LBD), indicating that specific ligands can be used to differentiate receptor-specific activity [34,70,74]. While the distribution of these receptors varies widely across different organs, both ERs are expressed in the breast tissue. Interestingly, while a consensus exists on ERα’s ability to promote breast tumorigenesis [75], conflicting reports have emerged regarding ERβ’s role as pro- or anti-tumorigenic [76,77,78,79,80,81,82,83,84]. Several reports indicate that ERα and ERβ have different transcriptomic overlap based on whether they exist as mono-receptors or are co-expressed in the tissue of interest [85,86,87]. Studies from our laboratory (Mukhopadhyay et al., manuscript in revision), and others have reported context-dependent pro/anti-tumorigenic functions of ERβ in breast cancer [83,88,89,90]. This apparent duality of ERβ function has very important therapeutic implications, especially in cancers such as triple negative breast cancer that do not express the ‘druggable’ ERα.

Similar to ERα, ERβ has also been shown to regulate metabolism. After ovariectomizing (Ovx) ERα knock out (ERαKO) mice, there was a decrease in body and fat pad weight as well as adipocyte circumference compared to sham-ovariectomized ERαKO mice. OvxERαKO mice also showed a reduction in insulin intolerance and increased glucose metabolism. All the observed metabolic differences between the ovx ERαKO and the sham-ovx ERαKO mice were reversed on treatment with E2. The authors hypothesized that ovariectomizing ERαKO mice removed the E2/ERβ signaling cascade and hence the observed metabolic changes were an indirect consequence of ERβ functioning. These observations suggest ERβ and ERα had opposite effects on fat metabolism [91]. Another study found no change in GLUT4 expression in the adipose tissue when ERαKO mice were treated with Tamoxifen. However, Tamoxifen treatment in ERβKO mice substantially decreased GLUT4 expression indicating that ERα was the major regulator of GLUT4 expression in the adipose tissue [64]. These studies, along with others, suggest that regulation of GLUT4 by ERs is complex and tissue type-specific. 

Recent studies in malignant mesothelioma cells revealed that ERβ repressed the expression of succinate dehydrogenase B (SDHB) a critical component of complex II (C-II) of the electron transport chain. This led to a decrease in complex-II activity and subsequently impaired mitochondrial oxidative phosphorylation (OXPHOS) [92]. Another study [93], investigated the role of ERβ in regulating the metabolism of breast cancer stem cells (BSCs) and the potential therapeutic implications of targeting ERβ in these cells. While the BSCs were ERα-negative they did express ERβ protein which was elevated in tumor-derived mammospheres and stem cells compared to differentiated cells. On the metabolic level, ERβ activation by a specific agonist 2,3-*bis*(4-Hydroxyphenyl)-propionitrile (DPN) induced a glycolytic gene signature, increased lactate secretion in the growth media, and decreased oxygen consumption rates (OCR) that could be rescued by treating with an ERβ specific antagonist 4-[2-Phenyl-5,7-*bis*(trifluoromethyl)pyrazolo[1,5-*a*]pyrimidin-3-yl]phenol (PHTPP). Knocking down ERβ reduced the expression of glycolytic genes while increasing the expression of mitochondrial genes involved in OXPHOS. In this regard, both ERα and ERβ seem to enhance glycolysis while repressing OXPHOS.

#### 2.1.3. Role of p53 in Regulating Cancer Metabolism

p53 is one of the most extensively studied tumor suppressor gene. Its importance to cancer cell growth is underscored by the fact that it is one of the most frequently mutated genes across all cancers including breast cancer [94,95,96,97]. A summary of numerous elegant studies led to the conclusion that p53 mediates its tumor suppressive functions by regulating the expression of genes that activate cell cycle arrest, apoptosis and senescence [98]. A seminal study [99] demonstrated that p53 was able to suppress tumorigenesis as well as tumor growth even in the absence of these three key programs. By mutating three essential acetylation residues (p53^3KR/3KR^) p53’s ability to activate cell cycle arrest, apoptosis and senescence was attenuated while its ability to regulate metabolism and ROS production was intact. Moreover, p53^3KR/3KR^ mice did not exhibit early onset of tumorigenesis similar to p53 null mice. This suggested that the ‘non-classical’ functions of p53 such as regulation of metabolism and ROS production are critical for suppressing the early onset of spontaneous tumorigenesis. This study brought to light the importance of inhibiting metabolic transformation as an essential tumor suppressive function of p53. p53 has been reported to regulate the function of various metabolic pathways at multiple levels [100]. Broadly, wild-type p53 has been reported to suppress glycolysis and activate OXPHOS. Wild-type p53 has been shown to suppress glycolysis by repressing the expression of glucose transporters GLUT1, GLUT3, and GLUT4 [101,102]. It regulates the expression of several enzymes involved in the glycolytic pathway either directly or indirectly, for e.g., hexokinase 2 (HK2), phosphofructokinase 1 (PFK1), phosphoglycerate mutase (PGM), pyruvate dehydrogenase (PDH), parkin 2 (PARK2), and pyruvate dehydrogenase kinase (PDK2) [103,104,105,106,107]. Overall, p53 increases mitochondrial respiration through a number of mechanisms. It upregulates the expression of synthesis of cytochrome c oxidase 2 (SCO2), an enzyme involved in the electron transport chain [108]. Besides activating SCO2, p53 elicits multiple transcriptional programs promoting the expression of genes related to mitochondrial biogenesis [109] such as apoptosis-inducing factor (*AIF*) [110,111] and ferredoxin reductase (*FDXR*) [112]. Mitochondrial citrate transporter protein (CTP, encoded by *SLC25A1*) is activated by mutant p53 [113]. p53 promotes fatty acid oxidation (FAO) by upregulating the expression of carnitine palmitoyltransferase 1C (CPT1C), malonyl-coenzyme A decarboxylase (MCD), lipin 1 (LPIN1), and pantothenate kinase (PANK1) [114,115,116,117]. Wild-type p53 also upregulates the expression of glutaminase 2 (GLS2), that increases the conversion of glutamine to glutamate [118,119]. The increased glutamate subsequently fuels the TCA cycle. This pathway is essential for replenishing NADPH and glutathione (GSH) pools, crucial antioxidants that counter oxidative stress. On the contrary, mutant p53 has been shown to drive glycolysis through the activation of RhoA/ROCK/GLUT1 signaling cascade [101]. Mutant p53 has also been shown to repress the activation of AMPK thereby repressing catabolic activities such as FAO while promoting anabolic processes such as increased fatty acid synthesis [120]. However, there are context-dependent exceptions to the general observation that wild-type p53 activates OXPHOS whereas mutant p53 activates glycolysis. For example, wild-type p53 has been reported to transcriptionally activate muscle specific PGM in cardiac and skeletal muscle of rats, potentially increasing glycolysis [121]. Furthermore, different p53 mutants can have diverse effects on metabolism [122].

Wild-type and mutant p53 have opposite roles in regulating fatty acid metabolism. Wild-type p53 prevents the shunting of the glucose carbon towards anabolic pathways such as the pentose phosphate pathway (PPP) by binding to and inhibiting glucose-6-phosphate dehydrogenase (G6PD) [123]. The oxidative phase of the PPP is responsible for the generation of NADPH which is required for the synthesis of lipids and essential precursors for nucleotide biosynthesis. Unlike wild-type p53, mutant p53 fails to inhibit G6PD activity [123]. p53 has an important role in lipid metabolism, including enhancing fatty acid oxidation and inhibiting fatty acid synthesis. As mentioned earlier, wild-type p53 enhances lipid oxidation by inducing the expression of LPIN1 [116]. However, wild-type p53 can also repress transcription of SREBP1c (sterol regulatory element-binding protein 1c) that activates the expression of fatty acid synthesis enzymes, including FASN and ACLY. Wild-type p53 has been reported to negatively regulate the mTOR pathway and the PPP, two pathways activated in tumors that regulate fatty acid synthesis [123,124]. On the contrary, mutant p53 increases lipid synthesis through its interaction with SREBPs, a family of transcription factors on sterol gene promoters. In particular, one study reported that mutant p53 upregulated genes involved in the mevalonate pathway which was subsequently shown to be involved in mediating the malignant phenotype induced by mutant p53 [125]. Importantly, mevalonate kinase (MVK) impacts regulation of mutant p53 by preventing the ubiquitination of a subset of p53 mutants (conformational mutants). In the presence of MVK these p53 mutants bound DNAJA1 (heat shock protein-40 family member) and this interaction protected mutant p53 from being ubiquitinated by CHIP (C-terminus of Hsc70-interacting protein) E3 ubiquitin ligase [126]. The two studies mentioned above hint at a possible positive feedback loop between mutant p53 and mevalonate pathway activation, suggesting potential therapeutic benefits of statins in subsets of cancers harboring p53 mutations. Aromatase, an enzyme responsible for the biosynthesis of estrogen from testosterone, is also regulated by p53. It has been reported that wild-type p53 can transcriptionally activate aromatase and that loss of wild-type p53 impairs the aromatase activity and leads to lipid accumulation in the livers of p53^−/−^ mice [127]. Interestingly, a study reported p53 mutations in ~38% of aromatase inhibitor-resistant breast tumors [128]. Broadly, these studies depict the crosstalk between p53 and sterol (cholesterol) synthesis pathways. This regulation is particularly interesting given the role of estrogen signaling via ER in breast tumorigenesis and that the crosstalk between ER-p53 can have important consequences on the numerous pathways that these two proteins regulate. This will be described in greater detail in the subsequent section. 

#### 2.1.4. ER-p53 Crosstalk and its Implications in Breast Cancer Metabolism

**Figure 1:**
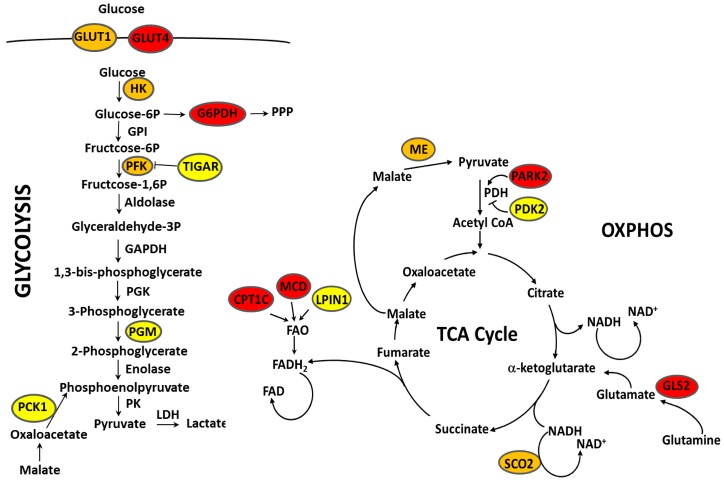
Metabolic crosstalk between ERα and p53. Metabolic targets regulated by both p53 and ERα are shown. These targets maybe regulated in the same direction (yellow: activated or repressed), opposite direction (red: repressed by one and activated by the other) or are bi-directionally regulated (orange: both activated and repressed).

Estrogen receptors and p53 play a critical role in mammary tumorigenesis, tumor maintenance and proliferation. Interestingly, these proteins have been reported to interact with each other with important functional consequences. Our studies have shown that ERα interacts with p53 and functionally antagonizes it [129,130,131]. Subsequently, transcriptional activation of ERα gene (*ESR1*) by p53 [132] and transcriptional repression of certain p53-target genes by ERα [133] have also been reported. More recently, studies from our laboratory (Mukhopadhyay et al., manuscript under revision) and others [88,89,134] have also shown that ERβ can interact with both wild-type and mutant p53 with different functional consequences. Functional antagonism of wild-type p53 by either estrogen receptor is detrimental, while that of mutant p53 is advantageous when considering tumor suppression. The primary read out of functional antagonism of p53 in these studies have been its classical tumor suppression functions, i.e., cell-cycle arrest, apoptosis, and senescence. However, it is now known that the tumor suppressive properties of p53 extend beyond these classical functions [99]. Questions remaining to be answered in this domain include: Can ERα bind mutant p53 and functionally antagonize it? Can p53 (mutant or wild-type) functionally antagonize ERα in certain cellular contexts? What are the implications of functional antagonism between ERs and p53 on their metabolic roles? Shown in Table 1 and Figure 1 are known p53 metabolic targets that are also regulated by ERα. The dual regulation of these genes by ERα and p53 suggest potential crosstalk, either cooperative or antagonistic. The molecular mechanisms underlying such crosstalk remain unclear.

### 2.2. Cell Extrinsic Cues and Metabolic Interactions with the Tumor Microenvironment

The role of the tumor microenvironment in altering the metabolism of tumor cells can be explained by the following classical example. As tumors proliferate rapidly, they outstrip their oxygen supply leading to a hypoxic environment. This in turn leads to the stabilization of HIF-1α which initiates the transcription of a number of enzymes involved in glycolysis while at the same time downregulating the expression of enzymes involved in the TCA cycle [151,152,153,154,155]. The switch from aerobic respiration to glycolysis would in part alleviate the hypoxic stress. However, this example explains only a part of the big picture as other molecular mechanisms play an important role in dictating the metabolic rewiring. 

**Figure 2:**
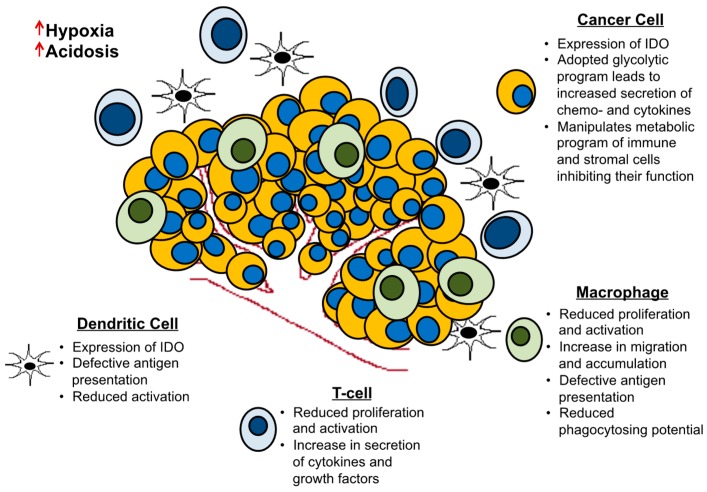
Metabolic interactions between the tumor and its microenvironment. T-cells, dendritic cells, and macrophages undergo metabolic reprogramming with different functional consequences (noted in the figure) that often propel tumor growth and progression.

As external cues, hypoxia, acidosis, and the extracellular matrix govern certain aspects of the metastatic and invasive phenotype of cancer cells (Figure 2). In order for cancer cells to metastasize they need to detach from the extracellular matrix (ECM) and suppress *anoikis* for survival during circulation in the blood or lymphatic system. Among other processes, detachment from the ECM can induce changes in metabolic pathways detrimental to the survival of cancer cells such as reduced glucose uptake, PPP flux, and cellular ATP levels while increasing the production of reactive oxygen species (ROS). In order to survive, the cancer cell must be able to counteract these fatal metabolic alterations, especially managing ROS levels. Studies have reported that upon detachment, normal mammary epithelial cells upregulate PDK4 via estrogen related receptor gamma thereby limiting the availability of the glucose carbon for mitochondrial oxidation, consequently suppressing *anoikis* [156]. Breast cancer cells on the other hand have inherent advantages of increased glycolysis and are hence able to survive in suspension. Stimulating PDH however, restores glucose oxidation and sensitizes the cells to *anoikis* while attenuating their metastatic potential [156]. Another way breast cancer cells counter increased ROS production is through the induction in expression of catalases such as manganese superoxide dismutase (MnSOD). Studies have demonstrated an increase in MnSOD expression in human breast cancer metastases compared to the primary tumor, while also reporting a positive correlation between MnSOD expression and tumor grade [157]. In an experimental metastasis model, where breast cancer cells were injected through the tail vein of immunocompromised mice, reduction in catalase levels resulted in a reduction in lung tumor burden [158]. Complimentary studies using a breast cancer mouse model have reported the importance of glutamate cysteine ligase modifier (GCLM) expression in increasing the production of endogenous antioxidants such as GSH for primary tumor formation. Loss of GCLM impaired the tumors ability to metastasize. Despite the threats posed by ROS, mitochondrial respiration is upregulated in circulating tumor cells compared to primary tumor cells [159]. It has been reported that proline dehydrogenase (PRODH) mediated proline catabolism is required for breast cancer cells grown in 3D culture. There was an increase in PRODH expression in metastatic compared to primary tumors in breast cancer patients as well as in a 4T1 mouse model. Targeting PRODH resulted in a decrease in lung metastases while sparing the normal tissue in the mouse model [160]. Changes in the density of extracellular matrix via collagen deposits also have a significant impact on the metabolic reprogramming of metastatic breast cancer cells [161]. When mouse mammary carcinoma cells were grown in high-density matrices, they displayed a reduction in utilization of the glucose carbon by the TCA cycle; instead the TCA cycle was fueled by glutamine. These functional changes were mirrored by changes in metabolic gene expression in the metastatic 4T1 cells.

Under conditions of metabolic stress such as hypoxia and nutrient deprivation, the enzyme acetyl-CoA synthetase 2 (ACSS2) enables the cancer cells to utilize acetyl-CoA as a source of carbon for lipid/biomass synthesis. There was a gain in copy number of ACSS2 in breast tumors and a positive correlation between its expression and disease progression [162]. Hypoxia leads to the stabilization of HIF-1α and the initiation of glycolytic transcriptional program. Lactate, the end product of glycolysis is released from the cell along with H^+^ ions with the help of monocarboxylate transporters and hydrogen ion pumps, causing extracellular acidification. This removal is crucial as accumulation of lactate and H^+^ ions in the cell would decrease the intracellular pH leading to cell death. The excess CO_2_ generated during mitochondrial metabolism is diffused into the extracellular space and subsequently converted into H^+^ and HCO_3_^−^ by carbonic anhydrases [163]. This reaction also leads to extracellular acidification, in turn stimulating the proteolytic activity of matrix metalloproteinases and the subsequent extracellular matrix remodeling, facilitating tumor invasion [164]. Extracellular lactate has also been reported to increase tumor invasion by stimulating the production of hyaluronic acid from the surrounding fibroblasts [165]. In addition, increased extracellular lactate induces the secretion of vascular endothelial growth factor (VEGF) by tumor associated stromal cells leading to increased angiogenesis [166].

The increase in extracellular lactate has implications on the immune system as well. Increased lactate levels provide tumor cells with an immune-conducive environment through the reduction in dendritic and T-cell activation [167]. Hypoxia and the extracellular acidification have also been reported to weaken immune cell function. Hypoxia facilitates the migration and accumulation of tumor-associated macrophages while at the same time inhibiting its phagocytosing potential. It allows for an increase in the release of cytokines and growth factors while preventing antigen presentation to T-cells [168,169]. Another way tumor cells suppress immune surveillance is by competing with T-cells for nutrients in the tumor microenvironment. T-cells originate from the lymphoid organs which are essentially nutrient replete. As they enter the harsh tumor microenvironment they are faced with several challenges that force a metabolic rewiring in order to survive, proliferate and function. Tumor cells can express indoleamine 2,3-dioxygenase (IDO) which inhibits T-cell proliferation by depleting tryptophan [170]. Aerobic glycolysis is required for optimal T-cell function but is not important for its proliferation or survival [171,172]. Tumor cells by restricting tumor infiltrating T lymphocytes (TILs) from undergoing aerobic glycolysis diminish their functional capabilities [173]. 

Tumor cells along with altering their own metabolic program affect the metabolism of stromal cells in their vicinity. Cells that form the core of the tumor and are spatially distanced from nutrient/oxygen rich vasculature, opt for a more glycolytic program than those located close to such vasculature. These cells release lactate which is taken up by the neighboring tumor/stromal cells and utilized as metabolic intermediates for biosynthesis or to meet their bioenergetics needs through OXPHOS [174]. It is estimated that about 80% of the breast cancer constitutes of stromal cells [175]. Cancer associated fibroblasts (CAFs) form a major component of the stromal cells. Tumor cells actively participate in rewiring the metabolic program of adjacent fibroblasts, converting them into cancer-associated fibroblasts. In co-culture experiments, MCF-7 cells induced oxidative stress and HIF-1α in adjacent fibroblasts, resulting in mitophagy and increased aerobic glycolysis. The lactate produced by the CAFs was then utilized by the tumor in a phenomenon known as the “reverse Warburg effect” [176,177]. Tumor suppressor genes such as BRCA1 may also affect the metabolic phenotype of stromal cells in the tumor microenvironment. HCC 1937 breast cancer cells harboring mutant BRCA1 induced oxidative stress and a glycolytic phenotype in co-cultured stromal fibroblasts. The altered stromal metabolic phenotype was caused by an increase in monocarboxylate transporter 4 (MCT4) and a loss in caveolin-1 (Cav-1) expression. The increase in expression was reverted when wild-type BRCA1 was overexpressed and caused the cancer cells to undergo apoptosis [26]. Similar to lactate, metabolomic profiling of CAFs revealed that they also produce glutamine, among other metabolites [178]. Supporting this data, it was subsequently demonstrated in co-culture experiments with MCF-7 cells that these cells displayed increased glutamine uptake transporters, increased glutamine catabolism, and reduced glutamine synthesis [179]. Fatty acids, another important metabolic fuel known to support tumor growth, have also been reported to be provided by the tumor microenvironment. Interestingly, tumor cells can also cause the transformation of adjacent adipocytes into “cancer associated adipocytes (CAAs)” [180]. As a toxic by-product of cellular metabolism, most cells dispose ammonia that is eventually degraded via the urea cycle. Tumor cells can acquire and assimilate ammonia present in its microenvironment giving it a growth advantage. The ammonia from the tumor microenvironment may be utilized by tumor cells as a nitrogen source for increased amino acid synthesis via increased glutamate dehydrogenase activity [181]. Another study involving both patient samples and in vitro co-culture experiments found that ovarian cancer cells adjacent to adipocytes accumulated both lipid droplets and an increased rate of FAO [182]. A similar observation was also noted in prostate cancer co-cultured with adipocytes. Incidentally, prostate cancer almost exclusively relies on FAO to meet its energy requirements [183]. As one can appreciate, with regards to altered metabolism, tumor cells may co-opt for a symbiotic relationship with stromal cells to enhance their growth and proliferation.

### 2.3. Cytoplasmic/Mitochondrial-Nuclear Crosstalk Regulates Breast Cancer Metabolism

A significant crosstalk through various feedforward and feedback loops occurs between the nucleus and cytoplasm/mitochondria in order to regulate cancer metabolism. Metabolites present in the cytoplasm/mitochondria may dictate DNA methylation and gene transcription while several transcription factors may shuttle between the nucleus and the mitochondria to transcribe genes that regulate metabolism. Mutations in isocitrate dehydrogenase 1 and 2 (IDH1 and IDH2) lead to a failure in decarboxylation of α-ketoglutarate (α-KG) to isocitrate, while increasing the conversion and accumulation of 2-hydroxyglutarate (2HG). Structurally similar to α-KG, 2HG acts as a competitive antagonist inhibiting the activity of α-KG dependent dioxygenases leading to global changes in DNA and histone methylation. This phenomenon occurs more commonly in gliomas and myeloid malignancies such as AML [184,185,186,187,188]. Interestingly, while the occurrence of IDH1/2 mutations in breast cancer is relatively rare, there have been mixed reports regarding its correlation with 2HG levels [189,190]. It was shown that high levels of 2HG were present even in the absence of IDH mutations and such high levels of 2HG correlated with MYC expression and glutamine consumption. Furthermore, tumors with elevated levels of 2HG were associated with increased DNA methylation and poor prognosis [191]. Similar to 2HG, succinate and fumarate too act as competitive antagonists to α-KG dependent dioxygenases, affecting DNA, and histone methylation. However, their role in breast cancer is relatively understudied and warrants further investigation. Another important metabolite involved in epigenetic regulation is acetyl-CoA. Histone acetyl transferase activity (HAT) and hence histone acetylation is regulated by the availability of acetyl-CoA. The levels of acetyl-CoA depend primarily on ATP citrate lyase (ACLY) and expression levels of its substrate, citrate. These expression levels maybe driven by oncogene mediated metabolic reprogramming. An in vivo study showed that induced expression of myristolated Akt (myrAkt) in mammary epithelial cells caused an increase in phospho-ATP citrate lyase (p-ACLY) levels as well as a pronounced increase in global histone acetylation [192]. Nuclear localization of ACLY and its phosphorylation by Akt facilitate the recruitment of BRCA1 and are essential for mediating DNA double strand break repair via homologous recombination [193]. DNA and histone methylation are important epigenetic modifications that are responsible for regulating access of the chromatin to the transcriptional machinery. These methylation events are carried out by DNA-methyltransferases (DNMTs) and histone methyltransferases (HMTs) that transfer methyl groups from a donor molecule. One of the major methyl group donors is S-adenosyl methionine (SAM), a derivative of methionine, an essential amino acid found in one’s diet. Methionine can also be formed by homocysteine via one carbon metabolism. Low levels of dietary folate (another methyl donor) have been reported to cause lymphocyte DNA hypomethylation in healthy postmenopausal women [194,195]. A similar correlation was observed between cervical tissue serum folate levels and DNA methylation [196].

On the other hand, epigenetic changes such as DNA methylation and histone modifications may also lead to changes in expression of genes involved in metabolism. For example, expression levels of Fructose 1,6 bisphosphatase 1 and 2 (FBP1 and FBP2) are silenced via promoter methylation. These enzymes are involved in gluconeogenesis and oppose glycolytic flux [197]. Increased expression levels of GLUT1 were regulated by promoter silencing of Derlin-3, a key gene involved in GLUT1’s proteasomal degradation [198]. The expression of the M2 isoform of pyruvate kinase (PKM2), another gene involved in glycolysis is regulated by promoter hypomethylation [199]. The promoters of genes such as PTEN, LKB1, and prolyl-hydroxylase domain family of enzymes (PHD1-3) are hypermethylated to “silence their suppression” of PI3K/AKT/mTOR and HIF-1α pathways which are known to drive glycolysis [200,201,202]. Histone modifications also play a role in regulating the metabolic phenotype. The sirtuin family of genes (SIRT) possessing histone deaceytlating activity has been extensively studied. One of the members, SIRT3, functions by destabilizing HIF-1α and consequently opposing its ability to induce glycolytic genes. However, its expression in tumor versus normal tissue and its implied metabolic consequence is debatable [203,204].

Additionally, metabolic enzymes may exhibit non-canonical (non-metabolic) functions which are critical for the survival and growth of cancer cells. As a metabolic enzyme, PKM2 plays an important role in regulating anabolic processes by diverting the glycolytic flux to the PPP and serine synthesis pathways (SSP). In MCF-7 cells, Jumonji C domain-containing dioxygenase (JMJD5) has been reported to bind PKM2. This interaction facilitates PKM2’s ability to translocate from the cytosol to the nucleus and enhance HIF-1α mediated transcriptional reprogramming [205]. Phosphoglycerate mutase 1 (PGAM1) responsible for the conversion of 3 phosphoglycerate to 2 phosphoglycerate has also been shown to modulate the cytoskeleton. In an in vitro breast cancer model, PGAM1 directly interacted with α-smooth muscle actin (ACTA2) and modulated actin filament assembly, cell mobility, and migration. Knocking down PGAM1 decreased the metastatic potential of breast cancer cells in vivo [206]. FBP1, a gluconeogenic rate limiting enzyme, converts fructose 1,6 bisphosphate to fructose 6 phosphate. Expression of FBP1 is repressed in a number of cancers including breast cancer [197]. In clear cell carcinoma, it has been reported that FBP1 binds to HIF-1α and represses its transcriptional activity [207]. While it has not been addressed directly, a similar inhibition of HIF-1α-mediated transcriptional regulation by FBP1 may exist in breast cancer. The transcriptional coactivators YAP/TAZ have been reported to interact with transcription factors from the TEA domain family to regulate gene transcription involved in survival and proliferation [208]. PFK1 has been shown to bind TEAD1 and 4 in the nucleus and is important for the stability of the YAP/TAZ/TEAD complex. Furthermore, overexpression of the allosteric activator of PFK1 has been shown to enhance YAP/TAZ mediated transcriptional activity [209].

Metabolite levels have also been recently shown to induce somatic mutations. Urea cycle dysregulation (UCD) has been seen across multiple cancer types including breast cancer. The authors of a recent study have quantified changes in enzymes involved in the urea cycle to generate a UCD score and reported a positive correlation between the UCD score of a tumor and its growth and proliferation. A high UCD score was shown to cause high pyrimidine to purine ratios and induce pyrimidine-rich transversion mutational bias in the sense strand of the DNA during transcription. This led to mutations in the DNA along with the presentation of neoantigens more susceptible to immune therapy [210]. These results also suggest that dietary differences might induce metabolite imbalances potentially contributing to disease risk [211]. Compartmentalization of metabolic intermediates and enzymes play a key role in modulating transcriptional regulation. The previously mentioned ACSS2 enzyme is one such example fulfilling distinct functions based on its cytosolic versus nuclear localization. A recent study showed that the utilization of exogenous acetate is predominantly controlled by ACSS2 and ACSS1 for lipogenic and mitochondrial purposes, respectively. On the other hand, in hypoxic regions of tumor, nuclear localization of ACSS2 is increased where it maintains histone acetylation by recapturing the acetate released from histone deacetylation for recycling by histone acetyl transferases [212].

The mitochondria produce bulk of the energy for normal cells. Only 13 of the 82 subunits that constitute the mitochondrial respiratory chain (MRC) are encoded by the mitochondrial DNA (mtDNA), while the remaining 69 subunits are encoded for by the nuclear genome. This indicates *a priori* the necessity of a potential crosstalk and functional coordination between the two genomes for the normal functioning of the mitochondria. Depleting breast cancer cells of their mtDNA resulted in altered expression of genes involved in growth, apoptosis, metabolism, cellular architecture, and differentiation [213]. An elegant study emphasized the role of mitochondria in tumorigenic and metastatic potential. Tumors formed from cells initially depleted of mitochondria, later acquired mtDNA from the host while recovering some respiratory functionality. The recovery of respiratory ability correlated with the metastatic potential indicating the importance of mitochondrial respiration in both tumorigenesis and metastasis [214]. Mutations in mtDNA, low mtDNA copy number, defects in the transcription or assembly of either nuclear or mitochondrially encoded MRC subunits may all contribute to dysfunctional mitochondria correlating with poor prognosis [215]. The development of transmitochondrial cybrid systems [216,217,218,219] provided another approach to understand the impact of the mitochondria on tumorigenesis, proliferation, and metastasis. In these systems, mitochondria from different cells varying in their proliferative or metastatic potential were fused individually with a common nucleus, hence avoiding the variability associated with the nuclear genome. Several studies have adopted this approach in addition to depleting the mitochondria to delineate the impact of the mitochondria on cell growth, proliferation, metastatic potential, as well as epithelial to mesenchymal transition (EMT) [214,219,220].

ERα and p53 as mentioned previously are intricately involved in the mitochondrial-nuclear crosstalk. While E2 stimulation of ERα has been reported to regulate the transcription of nuclear encoded mitochondrial genes, it remains unclear whether ERα can directly regulate transcription of mitochondrial genes via the mtDNA. ERα’s ability to induce the transcription of nuclear genes encoding mitochondrial components occurs via a multi-step process. One such mechanism is via the nuclear respiratory factor-1 (NRF-1) that binds its response elements present on the promoter of these genes, thus regulating their transcription. NRF-1 has also been reported to regulate the transcription of nuclear encoded mtDNA-specific transcription factor TFAM, thus integrating nuclear and mitochondrial signals [221]. E2 has been shown to increase the transcription of NRF-1 mRNA in breast cancer cell lines [222]. Hence, by increasing the transcription of NRF-1, E2 increases the transcription of TFAM and its mitochondrial target genes including COX IV and ND1 [221]. 

p53 activates transcription of TFAM by binding to the p53 response elements present on its promoter. Interestingly it has been shown that mutant p53 too can increase the expression levels of TFAM mRNA and protein leading to an increase in aerobic exercise capacity [223,224,225]. In addition to this, p53 has also been shown to regulate transcription of SCO2, one of the components of the electron transport chain. Lower levels of SCO2 lead to decreased OXPHOS [108]. Also, p53 is involved in maintaining the mitochondrial genome integrity along with its other non-metabolic functions; this topic has been extensively reviewed elsewhere [226].

## 3. Altered Metabolism in Primary versus Metastatic Lesions and Site-Specific Metabolic Alterations

One of the major challenges in cancer treatment deals with providing therapeutic options once the primary tumor has metastasized to distant organs. Disease prognosis drops drastically once metastasis has occurred and therapeutically recurrent primary and metastatic lesions are typically not sensitive to first line therapy. The metastatic process is complex and multifactorial in nature. Cancer cells in order to metastasize have to overcome a number of hurdles from intravasation to survival in the circulatory system and extravasation to a distant site. For solid tumors such as breast cancer, in order to survive anchorage independence, subsequent growth and proliferation in a foreign microenvironment presents a significant challenge. One of the ways cancer cells overcome these hurdles is by altering their metabolism. Several reports have looked into differences in metabolic reprogramming in primary tumors compared with their metastatic counter parts [4,5,6,7,8]. 

**Figure 3:**
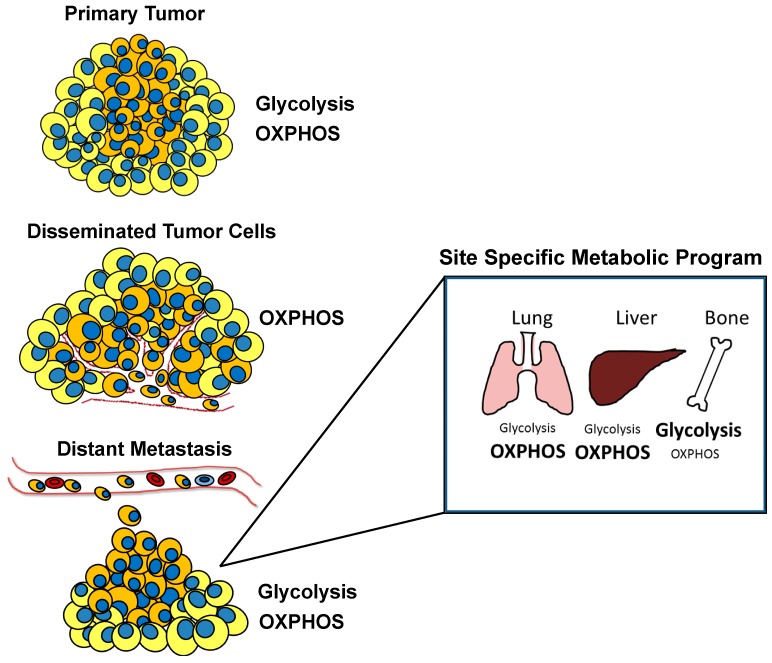
Metabolic plasticity of cancer cells during metastatic cascade. Cancer cells may adopt different energy pathways, i.e., glycolysis or OXPHOS based on their stage in the metastatic cascade as well as the site to which they metastasize.

A distinguishing feature between cancer cells with enhanced metastatic potential compared to non-metastatic cancer cells is their metabolic plasticity (Figure 3). This means that metastatic cancer cells do not overbearingly rely on a single metabolic pathway such as glycolysis or OXPHOS to meet their metabolic needs. These cells often run multiple metabolic pathways in parallel, regulating the utilization of the various pathways based on their adaptive needs as governed by cell extrinsic and intrinsic cues. A study investigating metabolic pathway differences between primary, circulating, and metastatic cancer cells in an isogenic mammary tumor background reported the importance of increased mitochondrial biogenesis and OXPHOS for cancer cells to metastasize. The authors found that several processes involved in the metastatic cascade such as invasion and migration favored mitochondrial respiration and ATP generation compared to anabolic pathways. It was reported that the transcription of PGC-1α was increased in circulating and metastatic cancer cells compared to the primary cancer cells [4]. Clinical data analysis showed correlation between the expression of PGC1-α in invasive cancer cells with their ability to form metastatic lesions. This study helped establish how a cell intrinsic cue was modified to meet the specific metabolic requirements of invasive and metastatic cancer cells. Another study utilizing dynamic magnetic resonance spectroscopy of radiolabeled carbon isotopomers reported an increase in glycolysis and OXPHOS in the highly metastatic 4T1 cells compared to non-metastatic 67NR breast cancer cells. This study also demonstrated that the metastatic cells displayed metabolic plasticity in that they were able to regulate flux between glycolysis and the TCA cycle depending on extrinsic cues [6]. A recurrent observation is that cancers originating from a particular histology preferentially metastasize to specific organs. Breast cancer has often been observed to metastasize to the lung, bone, liver, and brain. It has been hypothesized that the microenvironmental niche of these organs favors the metastasis of primary tumors to these sites. One might hence wonder if site-specific metastasis is governed by unique metabolic alterations. In a study using stable isotope tracer assay and microarrays, liver metastatic as compared to bone and lung metastatic breast cancer cells displayed an increase in glycolytic pathway and a reduction in glutamine metabolism and OXPHOS. The study also reported that PDK-1 was required to form efficient liver metastases and that the glycolytic phenotype observed in these cells was governed by both HIF-1α and PDK1 [5]. A previous study investigating metabolic alterations in brain metastatic breast cancer reported enhanced glycolysis coupled with mitochondrial respiration for energy production as well as increase in PPP. Interestingly, brain metastatic breast cancer cells were less susceptible to glucose deprivation as compared to parental or bone metastatic cells [7]. A recent study reported that increased levels of PGC1-α augmented the ability of breast cancer cells to metastasize to the lung and bone. This study argued the role of PGC1-α as a cell intrinsic cue responsible not only for promoting metastasis but also for playing a vital role in dictating the specific site of metastasis. The elevated expression levels of PGC1-α also provided metabolic flexibility to these cells, apart from increasing their respiratory capacity. Increased expression of PGC1-α facilitated the cells’ ability to opt for an alternate metabolic program when mitochondrial respiration was inhibited by biguanides such as metformin [8]. A similar study demonstrated that brain metastatic breast cancer cells were able to survive and proliferate independent of glucose by upregulating glutamine and branched chain amino acid oxidation [227]. As mentioned earlier, either local or distant recurrence poses a significant treatment challenge. Recurrence arises from cancer cells that manage to survive treatment insults and is broadly termed as minimal residual disease (MRD). A study investigating the metabolic changes accompanied with MRD reported gene expression pattern depicting altered lipid metabolism along with elevated ROS levels. Interestingly, they found that inhibiting either lipid synthesis or transport for oxidation resulted in reduced ROS levels and DNA damage. This study provided rationale for targeting lipid metabolism in MRD warranting further mechanistic and clinical evaluation [228].

## 4. Effects of Treatment and Resistance to SOC on Metabolic Reprogramming

Response to SOC and development of resistance to it are dependent on the specific subtype of breast cancer targeted.

### 4.1. Luminal A/B Subtype

Luminal breast cancer has been shown to exhibit an intermediate metabolic phenotype in the glycolysis-OXPHOS spectrum. Compared to TNBC these cancers are more reliant on OXPHOS [229]. As mentioned earlier, based on the amount of glucose available, E2 can promote glycolysis preferentially as compared to OXPHOS. Under conditions of high glucose E2 promoted glycolysis while in the case of low glucose it promoted OXPHOS [67]. This depicts an example of how external cues, in this case nutrient availability, can affect metabolic reprogramming. Nuclear magnetic resonance spectroscopy and proton magnetic resonance imaging showed that E2 induced glycolysis while Tamoxifen reduced it [230,231]. The induction of glycolysis by E2 and its reduction by tamoxifen were replicated in an orthotopic mouse model. This regulation was mediated via GLUT1 that was transcriptionally upregulated by E2 [232]. It has also been shown that E2 induces membrane-initiated rapid activation of the PI3K-Akt pathway as well as the transcriptional upregulation of 6-phosphofructo-2-kinase (PFKFB3) [233,234]. Both of these studies reveal additional therapeutic targets to be used in conjunction with hormonal therapy. Based on hormone receptor status; ER+ tumors had lower levels of glycine, choline, lactate, and glutamate (high glutamine) compared to ER-tumors. Low glutamate to glutamine ratio correlates with lower levels of glutaminolysis observed in ER+ tumors compared to TNBC [235].

Resistance to endocrine therapy occurs through multiple mechanisms. An increase in HER2 levels has been identified in tamoxifen resistant cells (TAM-R). One of the factors responsible for the increased HER2 levels is its post-transcriptional regulation by miR-26a/b and the RNA-binding protein human antigen R (HuR) [236]. Increased HER2 has been shown to regulate glycolysis indicating a potential role of dysregulated glycolysis in TAM-R cells. Increased glycolysis has also been observed in TAM-R cells via the activation of Akt/mTOR/HIF-1α axis. Inhibiting glycolysis via hexokinase II inhibition decreased Akt/mTOR/HIF-1α signaling and restored Tamoxifen sensitivity in antiestrogen-resistant breast cancer cells [237]. A study corroborating an increase in glycolysis in TAM-R cells also observed a counter intuitive increase in NRF-1 and its target TFAM [238]. Similarly, RNA sequencing studies to identify genes differentially expressed in tamoxifen-resistant vs. -sensitive cells found gene expression pattern suggesting dysfunctional mitochondria and altered OXPHOS in TAM-R cells [239]. Upregulation of these factors would typically increase mitochondrial activity via an increase in mitochondrial biosynthesis. The miR-29 transcriptome has also been shown to have differential effects in endocrine sensitive vs. resistant breast cancer cells. miR-29 has been shown to suppress growth of TAM-R cells in part by repressing genes involved in mitochondrial bioenergetics [240]. MYC has been found to be overexpressed in AI resistant breast cancer through the crosstalk between ER and HER2 pathways. Glutamine transporter SLC1A5 and GLS was significantly upregulated in AI resistant breast cancer cells and the inhibition of MYC, SLC1A5, and GLS was found to decrease the proliferation of AI resistant cells [241]. Hormonal therapy resistant metastatic cancer displays characteristics of enriched CD133, IL6 signaling, and low levels of ER. CD133^hi/^ER^lo^ leads to a suppression of mitochondrial OXPHOS [242]. A recent study reported a critical role of branched chain amino acid transaminase 1 (BCAT1) in antiestrogen-resistant and ERα- breast cancers. BCAT1 plays a major role in catabolizing the reversible transamination of branched chain amino acids into branched chain keto acids that might serve as substrates for TCA cycle activity [243]. Another study reported an increase in cholesterol/lipid and nucleotide metabolism in tamoxifen-resistant breast tumors. The authors from this study reported MUC1-induced lipid metabolism-related gene alterations, generating a gene signature predictive of response to tamoxifen treatment [244].

### 4.2. HER2-Enriched Breast Cancer

HER2+ breast cancer has been reported to exhibit a glycolytic phenotype. HER2 mediated signaling promotes glucose utilization, regulates lactate dehydrogenase-A (LDH-A) levels, PFKFB3 levels, and lactate accumulation in tumors [245,246,247,248,249]. In addition to these mechanisms, it has been reported in both cell lines and patient samples that HER2 translocates to the mitochondria in association with mitochondrial heat shock protein-70 (mtHSP70). Mitochondrial HER2 negatively regulates complex activity and oxygen consumption indirectly promoting glycolysis [250]. A novel EGFR/HER2 inhibitor KU004 was reported to decrease cancer cell proliferation via reduction in glycolysis [251]. Inhibition of PFKFB3 a critical enzyme involved in glycolysis suppressed growth of HER2 driven cancers. Using a mouse model driven by HER2 activation and LKB1 loss it was identified that therapies targeting mTORC1/mTORC2 and glycolysis provide the best therapeutic outcome [241]. Significantly higher expression of carnitine palmitoyltransferase-1A (CPT1A), perilipin-1(PLIN1), fatty acid synthase (FASN) have been reported in HER2+ tumors and cell lines compared to other subtypes [252]. It has also been observed that HER2+ tumors exhibit elevated levels of glycine, glutamine, succinate, and creatinine while displaying a reduction in alanine levels as compared to HER2- tumors [235].

Trastuzumab resistant cells have been reported to exhibit increased glycolysis. Glycolytic inhibition sensitized trastuzumab resistant (and responsive) HER2+ breast cancers to trastuzumab treatment [253]. Several mechanisms have been attributed to trastuzumab resistance in HER2+ breast cancer. One study identified increased levels of the truncated form of the dopamine and cAMP-regulated neuronal phosphoprotein 32 (t-Darpp) that interacts with the insulin growth factor receptor (IGF-1R) stimulating activation of IGF-1R signaling and glycolytic capacity. Hence t-Darpp was identified as a potential target inhibiting IGF-1R signaling and increased glycolysis in trastuzumab resistant HER2 cancer [254]. In a similar study, citrate therapy was shown to inhibit the phosphorylation of IGF-1R. By inhibiting the IGF-1R-Akt-PTEN-peIF2 pathway citrate therapy inhibited an inducible Her2 driven breast cancer model via the inhibition of both the TCA cycle and glycolysis [255]. Another study reported Skp2 SCF complex as the E3 ubiquitin ligase responsible for HER2 mediated Akt ubiquitination. Akt signaling cascade has been reported to increase glucose uptake and glycolysis via increased GLUT1 transcription and membrane translocation. While targeting glycolysis via Skp2, this study identified a means to sensitize HER2+ tumors to trastuzumab treatment [256]. The neuropeptide neuromedin U (NmU) has been shown to be increased in trastuzumab resistant HER2+ cancers. Ectopic expression of NmU has been shown to increase glycolysis likely via PDK activity positing as a potential therapeutic target [257].

In addition to the reliance of HER2+ breast cancers on glycolysis, these cells are also dependent on fatty acid synthesis, increased levels of stored fats and disruption of synthetic processes. In the presence of palmitate, FA synthesis was inhibited and glutamine, glucose, and serine/glycine metabolism was significantly altered via the activation of AMPK [258]. High levels of EphA2 in HER2+ tumors increase its dependency on glutamine metabolism via increase in transcription of glutamine transporter SLC1A5 and GLS identifying a novel therapeutic target in EphA2 [259]. These studies reflect the myriad of mechanisms involved in resistance to SOC and how metabolic intervention can be used to re-sensitize resistant cells to SOC. 

### 4.3. TNBC/Basal-Like-Cancer

The triple negative/basal-like subtype of breast cancer currently lacks therapeutic targets. Coupled with an inherently aggressive phenotype, TNBC often have a poorer prognosis compared to the other subtypes. TNBC has been reported to have a greater glycolytic phenotype as compared to luminal breast cancer based on their high extra cellular acidification rate (ECAR)/OCR ratio [229]. Other studies have reported increased glycolysis in TNBC via EGF signaling and that c-MYC drives glucose metabolism by suppressing thioredoxin-interacting protein (TXNIP)—an inhibitor of glycolysis [260]. In patients, the standard chemotherapy regimen in the adjuvant and neoadjuvant setting is sequential anthracycline-taxane based that maybe supplemented by platinum-based chemotherapy in some cases. Other regimens include combinations of adriamycin-taxane-cyclophosphamide, and 5-fluorouracil-epirubicin-cyclophosphamide followed by taxanes [261]. Combining doxorubicin with metabolic inhibitors such as metformin and sodium oxamate maximized tumor growth inhibition as compared to any bi-drug combination [262]. The expression of transketolase (TKT), a metabolic enzyme involved in the non-oxidative branch of the PPP also linking it to glycolysis, was found to correlate with tumor size in a syngeneic TNBC murine model. Pharmacological inhibition of this enzyme by oxythiamine treatment sensitized TNBC cells to doxorubicin or docetaxel [263]. The plant flavonoid luteolin, has been reported to reduce glycolytic flux and sensitize mouse mammary 4T1 cells to doxorubicin treatment [264]. Doxorubicin resistant breast cancers exhibit features of enhanced glycolysis as well as an increase in biosynthetic pathways. Using orthotopic mouse models it has been reported that targeting glycolysis made doxorubicin-resistant tumors sensitive to doxorubicin treatment. Others have reported that repressing phosphoglycerate dehydrogenase (PHGDH) sensitizes TNBC to doxorubicin [265]. Another study reported an increase in glucose metabolism in doxorubicin resistant cells mediated via an increase in FGFR4 expression levels. Inhibition of the FGFR4-Erk pathway led to a reduction in glucose metabolism and increased chemosensitivity [266].

Illustrating the metabolic plasticity of TNBC, two recent reports highlight the importance of OXPHOS in TNBC via an increased susceptibility to fatty acid oxidation inhibitors. In one study the authors showed that the tumors expressing high levels of MYC were susceptible to fatty acid oxidation while in the other study the authors showed that TNBC displayed elevated levels of fatty acid oxidation and activation of Src. Significant heterogeneity in metabolic adaptation exists even within TNBC [267,268]. Metabolic profiling of different subtypes of TNBC revealed differential susceptibility to metabolic modulators [269]. In terms of metabolites, TNBC showed a decrease in glutamine and an increase in choline and glutamate levels compared to triple positive breast cancer (TPBC). Another study in tumors from African American women reported a similar increase in glutaminolysis in TNBC compared to ER+ tumors. They also reported an increase in succinate, fumarate, and isoleucine indicating potential increase in TCA activity in TNBC compared to ER+ tumors [270]. These studies while highlighting metabolic plasticity within TNBC also suggest multiple potential adjuvant therapeutic targets. 

## 5. Current Status of Metabolic Intervention in Breast Cancer: Challenges that Lie Ahead

The past decade has seen a massive resurgence in the field of altered metabolism and targeting such pathways with a number of drugs in different stages of preclinical and clinical development (Table 2). However, the extremely promising preclinical studies have had at best modest clinical benefits. One of the major reasons for this stems from metabolic plasticity. This implies that drugs targeting specific metabolic pathways have an increased likelihood of failure owing to the ability of tumor cells to bypass the targeted pathway. This problem is often compounded by intra-tumoral heterogeneity, interactions with the tumor microenvironment and metastases. Another problem stemming from metabolic monotherapy and metabolic plasticity is that doses required to elicit an adequate response are often high and lead to unacceptable toxicities [271,272,273]. Hence, drugs fail due to insufficient dose and/or exposure to the tumor. To combat these problems, a multi-pronged approach targeting multiple metabolic pathways is warranted. In some cases, this can be achieved by using two or more drugs targeting different metabolic pathways. More importantly, in some cases one could leverage of the impact of standard of care therapies on altered metabolism, complimenting it with a drug targeting a compensatory metabolic pathway. Another approach to increasing the efficacy of metabolic therapy is by stratifying patients based on their genetic susceptibility to a specific intervention. Biguanides such as metformin and phenformin have been shown to be more efficacious in p53 null cells compared to wild-type p53 harboring cells [274]. 

Currently, one of the most attractive therapeutic opportunities is targeting tumors harboring mutations in IDH1/IDH2. This has been proven to be especially successful in hematologic malignancies [275]. Similarly, auxotrophies created as a result of the genetic make-up of certain tumors allow for a favorable therapeutic opportunity. For example, drugs that deplete arginine or serine in ASS1-deficient or p53 mutant tumors may prove to be successful candidates for clinical intervention [276].

## 6. Concluding Remarks

Altered metabolism as a therapeutic vulnerability is as promising as it is challenging. Metabolic plasticity need not be inherent but may be adaptive, based on the stage of tumor progression, metastatic site, and the type of treatment administered. Another layer of complexity arises from cell extrinsic cues such as nutrient availability; hypoxia and extracellular pH levels that often dictate the metabolic program of a cancer cell. The tumor microenvironment is often modified by tumor cells to aid in their metabolic reprogramming facilitating their growth and proliferation in a symbiotic manner. Information from these active areas of research should help in discovering new therapeutic strategies and repurposing existing drugs for effective intervention. Effective metabolic therapy would require precision medicine with integration of genomics, proteomics and metabolomics, stratifying patients into groups likely to benefit from specific intervention. 

## Figures and Tables

**Table 1:** List of genes and proteins regulated by both ERα and p53.

Metabolic Targets of p53	Regulation by p53	p53 Status ^∗^	Regulation by ERα	p53 Status ^∗^	References
G6PD	Repression	wt	Activation	wt	[135,136]
SREBP1	Repression	wt	Activation	wt	[136]
			Repression	wt	[135]
PFK1(PFKM)	Repression	wt	Repression	wt	[135]
			Activation	wt	[137]
PGM1	Repression	wt	Repression	wt	[136,138]
PDK2	Repression	wt	Repression	wt	[135]
PCK1	Repression	wt	Activation	wt, R280K	[139,140]
HK2	Repression/Activation	G103S, E256G	Repression	wt	[141,142]
			Activation	wt	[136,138,143]
GLUT1(SLC2A1)	Repression	wt	Repression	wt	[136,142,144]
			Activation	wt, R280K, E285K	[135,138,140,143,145,146,147]
GLUT4(SLC2A4)	Repression	wt	Activation	wt	[135,148]
ME1	Repression	wt	Repression	wt, L194F, E285K	[135,138,149]
			Activation	wt	[136,139,141]
ME2	Repression	wt	Repression	wt	[136]
			Activation	wt	[138]
TIGAR	Activation	wt	Activation	wt, L194F, E285K	[149]
PARK2	Activation	wt	Repression	wt	[150]
SCO2	Activation	wt	Activation	wt	[144]
			Repression	wt	[138]
LPIN1	Activation	wt	Activation	wt	[150]
CPT1C	Activation	wt	Repression	wt	[136]
GLS2	Activation	wt	Repression	wt	[135]
MCD (MLYCD)	Activation	wt	Repression	wt	[136]

^∗^ p53 status of cell lines used to determine the ability of p53 and ER to regulate the metabolic enzymes listed in the table. wt: wild-type.

**Table 2:** Drugs currently in different stages of development as metabolic interventions in breast cancer

Target Protein and Pathway	Drug	Type	Indications	Clinical Trials
**Glycolysis**				
SGLT-2	Dapagliflozin	Retrospective/Observational	Incidence of breast and bladder cancer	NCT02695121
Hexokinase	2-deoxy-D-glucose (2DG)	Phase I	Breast cancer and advanced solid malignancies	NCT00096707
TCA Cycle and MRC				
PDK	Dichloroacetate	Phase II	Metastatic breast cancer or NSCLC	NCT01029925
PDH/KGDH	CPI-613	Phase II	Advanced solid tumors	NCT01832857
	ME-344	Early Phase I	Her2- metastatic breast cancer	NCT02806817
Complex I	Metformin	Phase I/Phase II/Phase III	All breast cancer	multiple clinical trials
**Lipid Synthesis**				
FASN	TVB-2640	Phase II	Her2+ metastatic breast cancer resistant to trastuzumab and taxanes	NCT03179904
	Omeprazole	Phase II	Triple negative breast cancer	NCT02595372
	Conjugated Linoleic Acid (CLA)	Phase I	Metastatic breast cancer	NCT00908791
**AA Metabolism**				
Glutaminase	CB-839	Phase I/Phase II	Advanced solid tumors/advanced TNBC	NCT02071862 NCT03057600
Indoleamine 2,3 dioxygenase (IDO1)	Indoximod	Phase I/Phase II	Metastatic breast cancer	NCT01792050 NCT01042535
	Epacadostat	Phase I/Phase II	TNBC and other selected cancers	NCT02178722
Arginine deiminase (ADI)	ADI-PEG20	Phase I	Her2- metastatic breast cancer	NCT01948843
